# Impact of diabetes mellitus on spinal injection two-week outcomes in patients with predominant lower extremity pain

**DOI:** 10.1007/s00256-025-04979-2

**Published:** 2025-07-10

**Authors:** Youngjune Kim, Choong Guen Chee, Yusuhn Kang, Eugene Lee, Joon Woo Lee

**Affiliations:** 1 Department of Radiology, Seoul National University Bundang Hospital, Seongnam, South Korea; 2 Seoul National University College of Medicine, Seoul, South Korea

**Keywords:** Diabetes Mellitus, Radiculopathy, Treatment outcome, Spinal injection

## Abstract

**Objective:**

To evaluate the effect of diabetes mellitus (DM) on the effectiveness of spinal injections in patients with predominant lower extremity pain.

**Materials and methods:**

This retrospective study included 218 patients (109 with DM and 109 without DM, matched by propensity scores; mean age 68.4 ± 9.6 years; 111 men) who underwent lumbar spinal injections in 2017. Treatment effectiveness was assessed by calculating the relative reduction in numerical rating scale (NRS) scores 2 weeks post-injection. A generalized estimating equation analysis was conducted to determine whether DM influenced overall treatment outcomes and to perform subgroup analyses based on age (< 50 years, ≥ 50 years), sex, pain onset (acute/subacute [≤ 6 months] vs. chronic [> 6 months]), preprocedural pain severity (mild [NRS 0‒3], moderate [NRS 4‒6], severe [NRS 7‒10]), and pain symmetry (symmetric vs. asymmetric). Fisher’s exact test was used to compare adverse event rates between patients with and without DM.

**Results:**

DM did not significantly impact the overall effectiveness of spinal injections 2 weeks after the injection (relative NRS reduction difference [95% confidence interval], –5.5% [–13.3% to 2.2%]; P = 0.163). However, patients with DM and chronic pain (–16.7% [–25.6% to –7.9%], P < 0.001) or mild preprocedural pain (–20.2% [–37.1% to –3.2%], P = 0.019) exhibited reduced treatment effects 2 weeks after the injection. Adverse event rates did not significantly differ between groups (P > 0.99).

**Conclusion:**

There was no difference in the spinal injection effectiveness between patients with and without DM 2 weeks after the injection. However, their therapeutic effect may be diminished in patients with chronic or mild preprocedural pain, warranting consideration of a potential diagnostic overlap with diabetic neuropathy.

## Introduction

Diabetes mellitus (DM) is becoming increasingly prevalent in the United States, affecting approximately 38.4 million individuals (11.6% of the population) [1]. This rise in diabetes cases spans various social, economic, and ethnic backgrounds, posing a significant public health concern. As diabetes becomes more widespread, its implications for treatment outcomes across various medical interventions, including pain management strategies, have been extensively investigated.

Diabetic neuropathy is a common complication of DM, causing pain in up to 34% of patients with diabetes [2]. Among the different types of diabetic neuropathy, diabetic peripheral neuropathy (DPN) is the most prevalent. DPN can lead to chronic pain, paresthesia, and sensory deficits, particularly in the lower extremities [3]. At the same time, lumbar spine disorders‒such as herniated intervertebral discs and spinal stenosis‒can produce similar symptoms. This overlap in clinical presentations complicates diagnosis, making it challenging for clinicians to accurately identify the primary source of pain or assess the relative contribution of each condition to a patient’s symptoms [4].

Moreover, growing evidence suggests that DM is associated with an increased risk of degenerative lumbar spine disorders [5]. This association adds diagnostic complexity, as DM may not only contribute to neuropathic symptoms but also exacerbate or accelerate lumbar spine pathology. Additionally, the microvascular changes caused by DM can alter the local tissue environment and drug distribution. Furthermore, diabetic polyneuropathy may affect a patient’s response to local anesthetics and corticosteroids commonly used in spinal interventions, potentially influencing the efficacy of spinal injections [6, 7].

We aimed to determine whether DM affects the effectiveness of spinal injections, particularly in patients with predominant lower extremity pain. By comparing cohorts of patients with and without DM who underwent lumbar spinal injections, we sought to elucidate the relationship between DM and treatment outcomes.

## Materials and methods

This retrospective study was approved by our institutional review board (no. B-2412–942-108). Due to the retrospective nature of the study, the requirement for written informed consent was waived.

### Patient inclusion

In 2017, 2106 patients initially visited our spinal intervention center and underwent lumbar spinal injections for predominant lower extremity pain. All patients who received injections either demonstrated lesions on lumbar spine MRI, which can potentially cause lower extremity pain, or were clinically suspected of having spine-origin pain, leading to referrals from neurosurgeons or orthopedic surgeons for spinal injections. Two fellowship-trained attending radiologists (with 14 and 6 years of experience in spine radiology and intervention) and five musculoskeletal fellows reviewed all lumbar spine MRIs during patient interview prior to the spinal injections and performed the spinal injections. Patients were excluded based on the following criteria: i) equivalent pain intensity in both the axial and lower extremity regions, ii) predominant axial pain, iii) inability to provide post-intervention numerical rating scale (NRS) scores during the 2-week follow-up telephone interview, and iv) incomplete documentation of preprocedural pain intensity using NRS scores. Although some patients from this study were included in our previous publications [8–10], the present investigation addresses an entirely different research question and provides novel analyses not explored in our prior work.

### Patient information

At their initial outpatient clinic visit, all patients were asked to provide the following information using a structured questionnaire: i) presence or absence of DM, ii) pain location (predominant lower extremity pain, predominant axial pain, or equal pain intensity in both regions), iii) pain onset (acute and subacute [≤ 6 months], chronic [> 6 months]), iv) preprocedural pain intensity documented using 11-point NRS, and v) pain symmetry (symmetric or asymmetric).

### Spinal injection

The choice of injection technique was based on the specific pathology detected on MRI, in conjunction with the clinical presentation. The radiologists performed the spinal injections under fluoroscopic guidance with patients in the prone position. Interlaminar epidural injection is typically used for central pathologies such as spinal stenosis or central herniated discs, while transforaminal epidural injection is preferred for subarticular, foraminal, or extraforaminal herniated discs. Caudal injection was considered when other approaches were unsuitable (e.g., multilevel pathology, lack of epidural fat). Intra-articular facet joint injections have been used for facet osteoarthritis, instability, and spondylolisthesis, though evidence supporting their efficacy is limited [11]. Recent studies suggest that facet joints may contribute to pain in osteoporotic vertebral compression fractures, supporting the use of facet injections in cases where severe pain persists despite conservative treatment and surgery is not indicated [12, 13]. In post-lumbar surgery syndrome, the injection technique was tailored to the specific pathology, which could include spinal stenosis, disc herniation, instability, spondylolisthesis, or facet joint involvement.

The dosage of the injectables and protocol for spinal injections were tailored for each procedure [8, 14]. A mixture of dexamethasone and ropivacaine was administered for lumbar interlaminar and transforaminal epidural injections. The interlaminar approach used 10 mg of dexamethasone with 7.5 mg of ropivacaine, while the transforaminal method used the same amount of dexamethasone but with 3.75 mg of ropivacaine. The lumbar facet joint injections consisted of a mixture of triamcinolone and ropivacaine. Each facet joint received 20 mg triamcinolone combined with 3.75 mg of ropivacaine. For caudal injections, a mixture of 10 mg dexamethasone and 3.75 mg ropivacaine was administered. The injection methods and dosages were consistent, regardless of the patient’s DM status.

### Outcome measure

A 2-week post-injection telephone interview was performed focusing on the treatment effect of the spinal injection and any systemic adverse events. The relative reduction in NRS was calculated based on the preprocedural NRS obtained during the initial outpatient clinic visit and the postprocedural NRS obtained during the telephone interview. Systemic adverse events were documented with specific inquiries regarding pain exacerbation, facial flushing, pruritus, insomnia, hyperglycemia, and gastrointestinal disturbances, as well as a broad inquiry regarding other symptoms.

### Statistical analysis

Patients with DM were matched to those without DM in a 1:1 ratio using propensity score matching. Propensity scores were calculated assuming a linear relationship between DM and baseline characteristics (i.e., age, sex, onset of pain, pain symmetry, preprocedural NRS score, and type of injection). Subsequently, optimal matching was performed to ensure the most suitable pairing of subjects based on propensity scores. To assess the balance of baseline characteristics between the two groups before and after matching, we calculated the standardized mean difference for continuous variables and the absolute mean difference for categorical variables. A standardized or absolute mean difference of less than 0.1 and a variance ratio less than 2 were considered to indicate that a particular baseline characteristic was balanced between the two groups.

A generalized estimating equation (GEE) analysis was performed to assess whether DM affected 2-week treatment outcomes. Additional subgroup analysis was performed using a GEE analysis to identify specific subgroups in which treatment outcomes might be affected by DM. The tested subgroups included: age (< 50 years, ≥ 50 years), sex, pain onset (acute and subacute [≤ 6 months], chronic [> 6 months]), preprocedural pain severity (mild [NRS 0–3], moderate [NRS 4–6], severe [NRS 7–10]), and pain symmetry (symmetric, asymmetric). Given that spinal pathology differs above and below 50 years of age, 50 years was selected as the age cutoff for subgroup analysis [8]. Fisher’s exact test was used to compare patient-reported adverse event rates between patients with and without DM. All statistical analyses were performed using R version 4.3.3 (The R Foundation for Statistical Computing, Vienna, Austria). A two-sided P-value < 0.05 was considered statistically significant.

## Results

**Fig. 1:**
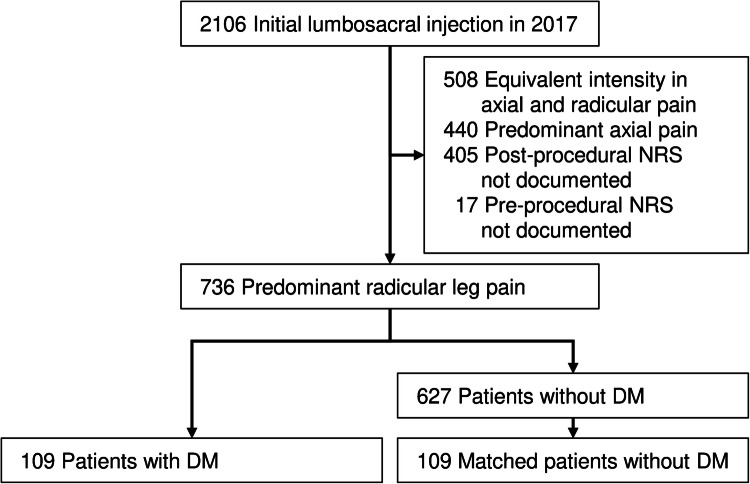
Patient inclusion diagram. NRS, numerical rating scale; DM, diabetes mellitus

Among 2106 patients who received lumbar spinal injections at our spinal intervention center in 2017, 1370 were excluded for the following reasons: 508 patients had equivalent pain intensity between the axial and lower extremities, 440 patients had predominant axial pain, 405 lacked postprocedural NRS documentation required 2 weeks after the procedure, and 17 had missing preprocedural NRS documentation (Fig. 1). The baseline characteristics of the remaining 736 patients are summarized in Table 1. After propensity score matching, we analyzed 218 patients (mean age ± standard deviation, 68.4 ± 9.6 years; 111 men), comprising 109 patients with DM and 109 matched patients without DM (Table 1). There were no statistically significant differences in baseline characteristics between the groups after matching.

**Table 1:** Baseline characteristics of injected patients before and after propensity score matching

		Patients with diabetes (n = 109)	Patients without diabetes	
			Unmatched (n = 627)	Matched (n = 109)
Age (year)—mean ± standard deviation		68.1 ± 9.2	61.6 ± 14.6	68.8 ± 10.0
Sex				
	Female	53 (48.6%)	354 (56.5%)	54 (49.5%)
	Male	56 (51.4%)	273 (43.5%)	55 (50.5%)
Onset of pain				
	≤ 6 months	47 (43.1%)	332 (53.0%)	40 (36.7%)
	> 6 months	59 (54.1%)	290 (46.3%)	67 (61.5%)
	Unknown	3 (2.8%)	5 (0.8%)	2 (1.8%)
Pain symmetry				
	Symmetric	49 (45.0%)	178 (28.4%)	45 (41.3%)
	Asymmetric	60 (55.0%)	449 (71.6%)	64 (58.7%)
Pre-procedure numerical rating scale score				
	Mild (0–3)	26 (23.9%)	148 (23.6%)	21 (19.3%)
	Moderate (4–6)	31 (28.4%)	170 (27.1%)	33 (30.3%)
	Severe (7–10)	52 (47.7%)	309 (49.3%)	55 (50.5%)
Injection type				
	Lumbar interlaminar	27 (24.8%)	142 (22.6%)	24 (22.0%)
	Lumbar transforaminal	41 (37.6%)	284 (45.3%)	45 (41.3%)
	Lumbar facet	13 (11.9%)	99 (15.8%)	13 (11.9%)
	Caudal	25 (22.9%)	86 (13.7%)	23 (21.1%)
	Others	3 (2.8%)	16 (2.6%)	4 (3.7%)

Data are numbers of patients and percentages, unless otherwise specified. Others in the injection type refers to lumbar injections that involved mixed techniques (e.g., combination of lumbar transforaminal and interlaminar methods)

**Fig. 2:**
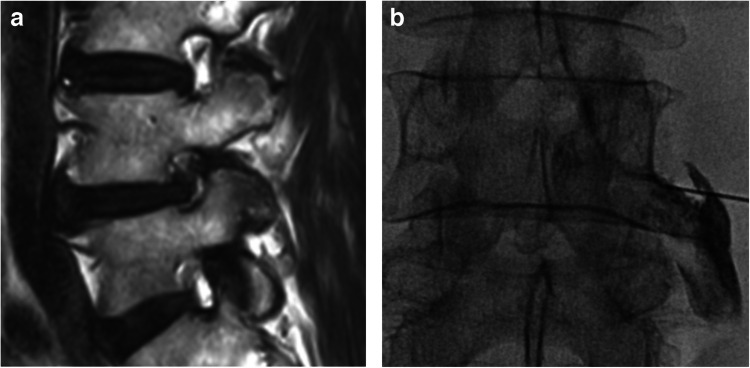
A 72-year-old male with diabetes mellitus presented with right leg pain started 10 years ago. **a** Lumbar spine MRI revealed severe foraminal stenosis at the right L4–L5 foraminal space, prompting a planned right L4–L5 transforaminal epidural steroid injection. **b** A fluoroscopy-guided right L4–L5 transforaminal epidural steroid injection was performed. The patient’s numerical rating scale score for pain was 5 before the injection and remained unchanged at 5 two weeks after the procedure

The GEE analysis revealed no statistically significant difference in the relative reduction in NRS between patients with DM and matched patients without DM (difference in NRS reduction two weeks after the injection [95% confidence interval (CI), –5.5% [–13.3% to 2.2%]; P = 0.163) (Table 2). However, subgroup analysis showed that DM was associated with a diminished 2-week treatment effect of spinal injections in patients with chronic pain (difference of NRS reduction [95% CI], –16.7% [–25.6% to –7.9%]; P < 0.001) and mild preprocedural pain (–20.2% [–37.1% to –3.2%], P = 0.019). In the other subgroups, no significant difference in 2-week treatment outcomes was observed between patients with and without DM. A representative case with DM is shown in Fig. 2.

**Table 2:** Subgroup analysis comparing treatment effects between patients with diabetes and matched patients without diabetes

			Patients with diabetes (n = 109)	Matched patients without diabetes (n = 109)	Difference in relative NRS reduction (95% CI)	P-value
Age						
	< 50 years		15.7% ± 13.7%	48.3% ± 38.4%	–30.6% (–64.1% to 2.9%)	0.073
	≥ 50 years		23.1% ± 29.1%	27.7% ± 28.3%	–4.6% (–12.5% to 3.3%)	0.255
Sex						
	Female		22.1% ± 25.2%	24.3% ± 25.0%	–2.2% (–11.0% to 6.6%)	0.630
	Male		23.8% ± 32.1%	32.6% ± 31.7%	–9.0% (–21.3% to 3.5%)	0.160
Onset of pain						
	≤ 6 months		33.0% ± 30.0%	25.3% ± 31.7%	8.6% (–4.7% to 22.0%)	0.206
	> 6 months		13.5% ± 23.1%	30.4% ± 26.9%	–16.7% (–25.6% to –7.9%)	< 0.001
Pain symmetry						
	Symmetric		20.2% ± 28.1%	29.8% ± 28.6%	–8.7% (–20.6% to 3.3%)	0.155
	Asymmetric		25.2% ± 29.4%	27.5% ± 29.1%	–2.2% (–11.9% to 7.6%)	0.665
Preprocedural NRS						
	Mild (0–3)		13.1% ± 26.2%	33.8% ± 31.5%	–20.2% (–37.1% to –3.2%)	0.019
	Moderate (4–6)		27.7% ± 33.3%	16.5% ± 25.5%	11.6% (–2.9% to 26.1%)	0.118
	Severe (7–10)		25.0% ± 26.5%	33.6% ± 27.9%	–9.0% (–18.9% to 0.9%)	0.076
Injection type						
	Lumbar interlaminar		19.5% ± 28.7%	25.7% ± 27.7%	–8.6% (–24.9% to 7.8%)	0.306
	Lumbar transforaminal		23.1% ± 29.4%	24.6% ± 28.8%	–3.7% (–15.4% to 7.9%)	0.529
	Lumbar facet		30.8% ± 38.8%	42.6% ± 25.2%	–12.8% (–37.1% to 11.6%)	0.304
	Caudal		21.3% ± 21.7%	27.1% ± 30.0%	–5.3% (–20.0% to 9.3%)	0.475
Total			22.9% ± 28.8%	28.5% ± 28.8%	–5.5% (–13.3% to 2.2%)	0.163

Data are presented in mean ± standard deviation of relative NRS reduction, unless otherwise specified. CI, confidence interval; NRS, numerical rating scale

Patient-reported adverse events are listed in Table 3. Most patients with DM (91.7%, 100/109) and those without DM (90.8%, 99/109) reported no adverse events. There was no statistically significant difference in adverse event rates between groups (P > 0.99).

**Table 3:** Patient-reported adverse events between patients with and without diabetes mellitus

	Patients with diabetes (n = 109)	Matched patients without diabetes (n = 109)
None	100 (91.7%)	99 (90.8%)
Pain exacerbation	3 (2.8%)	5 (4.6%)
Facial flushing	2 (1.8%)	0 (0.0%)
Pruritus	1 (0.9%)	1 (0.9%)
Insomnia	0 (0.0%)	2 (1.8%)
Hyperglycemia	2 (1.8%)	0 (0.0%)
Gastrointestinal trouble	1 (0.9%)	0 (0.0%)
Others	1 (0.9%)	3 (2.8%)

Data are presented as the number of patients with percentages in parentheses. Some patients reported two or more symptoms; therefore, the total sum of numbers did not match the total number of patients, and thus, the sum of percentages did not equal 100

## Discussion

We evaluated the effect of DM on the efficacy of spinal injections in patients with predominant lower extremity pain. Our findings indicate that DM did not diminish the overall effectiveness of lumbar spinal injections 2 weeks after the injection, as evidenced by comparable NRS reductions between patients with DM and matched patients without DM. However, subgroup analyses revealed a diminished 2-week treatment effect in patients with DM who had chronic pain and mild preprocedural pain. There was no difference in adverse event rates between the groups.

Our findings demonstrated that DM did not significantly impair the overall effectiveness of lumbar spinal injections 2 weeks after the injection. Our findings align with those of previous research [6, 15], demonstrating that patients with diabetes benefit from epidural steroid injection to a similar extent as individuals without diabetes. This is particularly important given the complex interplay between DM and neuropathic pain, as well as concerns about the efficacy of interventional pain management in patients with metabolic disorders.

However, our subgroup analysis revealed a reduced treatment effect 2 weeks after the injection in certain DM subgroups, contrasting with previous studies [6, 15]. This highlights the importance of considering individual patient characteristics when evaluating the efficacy of spinal injections in patients with diabetes. Unlike Wong et al. [6] who analyzed all patients receiving epidural steroid injection regardless of symptoms, our study focused specifically on patients with predominant lower extremity pain, potentially caused by both spinal pathology and DPN. Ma et al. [15] examined patients who received transforaminal epidural injections for cervical radiculopathy. However, DPN primarily affects the distal nerves of the lower extremities rather than the upper extremities. Consequently, the effect of DM on radicular pain in the upper limbs may be less prominent. This distinction in patient selection may explain the differing results and emphasizes the need to consider pain site when evaluating the influence of DM on treatment outcomes.

Reduced efficacy has been observed in patients with DM and chronic pain or mild periprocedural pain. The diminished response in the chronic pain subgroup may be attributed to nerve damage from both spinal pathology and prolonged hyperglycemia, which likely induces irreversible axonal damage and glial activation. This process leads to metabolically driven neuropathic pain, rendering transient anti-inflammatory interventions less effective. Additionally, DPN often manifests as numbness, tingling, or burning sensations rather than pain. In cases of mild preprocedural pain, it is necessary to verify whether the symptoms are due to DPN rather than spinal pathology. This suggests that in patients with DM who complain of mild and chronic lower extremity pain, despite the presence of pathology on MRI that could potentially cause symptoms, the actual source of the patient's symptoms might be DPN rather than lumbar pathology [16, 17]. Alternatively, it is possible that both DPN and lumbar pathology contribute to the symptoms, leading to a significant reduction in treatment effectiveness. Therefore, in patients with diabetes presenting with lower extremity pain, our study highlighted that both lumbar pathology and diabetes management play critical roles in controlling symptoms. A comprehensive approach to pain management in patients with diabetes is needed, considering both spinal pathology and the potential contribution of diabetic neuropathy to the overall symptom profile.

Interestingly, symmetric lower extremity pain was not associated with decreased 2-week treatment outcomes in patients with DM. Although symmetric symptoms in patients with DM may reflect DPN, a considerable proportion of patients with central canal stenosis also exhibit symmetric lower extremity leg pain. This may explain why pain symmetry was not correlated with treatment outcomes.

The comparable adverse event rates between patients with and without DM reinforce the safety of spinal injections in diabetic populations. While studies have reported an association between DM and systemic reactions following epidural steroid injection [18] and between DM and perioperative complications following spinal surgery [19], our results showed no increased risk of adverse events following spinal injection in patients with DM. Although concerns exist regarding hyperglycemia exacerbation post-steroid injection [20–24], its clinical impact seemed to be minimal and transient in our patients. Nevertheless, as previous research has found that complicated DM is associated with deep spinal infection [25], it remains crucial to be vigilant for deep spinal infection in patients with DM, despite its low incidence following spinal injection [25, 26].

Our study had some limitations. First, the single-center retrospective design might have limited the generalizability of our findings. Second, owing to the retrospective nature of the study, we were unable to examine the exact serum glucose levels or HbA1c. As Wong et al. suggested that higher serum HbA1c levels may be associated with poorer outcomes [6], accurate documentation of HbA1c levels in the included patients would have been beneficial for a more comprehensive analysis. Third, there is a possibility of underreporting adverse events during the telephone interviews. Patients may have failed to disclose certain symptoms or may not have accurately recognized the symptoms of injection-related adverse events, potentially leading to an incomplete assessment of the safety profile of the procedure. Finally, as we evaluated treatment outcomes within a relatively short follow-up period after the injection, the impact of DM on long-term efficacy remains undetermined.

In conclusion, while DM did not affect the overall treatment effect of spinal injections, a decreased treatment effect was observed in subgroups with chronic pain and mild preprocedural pain. These findings highlight the importance of personalized treatment approaches and the need for careful patient selection when considering spinal injections for patients with diabetes.

## Data Availability

The dataset is available from the corresponding author upon reasonable request.

## Abbreviations

DM: Diabetes Mellitus
NRS: Numerical Rating Scale
DPN: Diabetic Peripheral Neuropathy
MRI: Magnetic Resonance Imaging
CI: Confidence Interval
HbA1c: Hemoglobin A1c
